# The Tait and Battey Controversy

**Published:** 1886-08

**Authors:** 


### THE TAIT AND BATTEY CONTROVERSY.

In The Journal for June we reproduced from the *Medical News* a letter of Dr. Robert Battey's regarding some statements by Mr. Lawson Tait as to his claims for priority in Battey's operation, with the following comment:

"The following letter from Dr. Robert Battey, in the *Medical News*, May 15th, touching an error in the statement of results of Battey's and Tait's operation, is calculated to impress upon American readers certain misgivings as to the trustworthiness of the reports made by Mr. Lawson Tait in regard to his gynecological work. If an operator varies his statement of results in given cases to suit his ulterior ends or to meet the issues presented by the experience of others, we have no safe guide to a discussion of any mooted question in the published data of such surgeon, and Mr. Tait should know that 'facts are stubborn things.'"

Since that time we have received a letter from Mr. Tait, which appears elsewhere, in which he claims The Journal has been unfair in its comments. He at the same time enclosed to us a copy of a letter he had forwarded to the *Medical News* in reply to the letter of Dr. Battey's on which our comments were made. We disclaim any intention of being unfair or doing an injustice to Mr. Tait, and we contend that our criticism was just and fair with the facts then before us. We want the truth and nothing but the truth. Below is Mr. Tait's last letter to the *Medical News* in full:

I am sorry that I have, inadvertently, as Dr. Battey says, stated that Dr. Battey's first case had a fatal result. That the mistake was a slip of the pen, or a mistake in dictating to my secretary is certain, because, at page 326 of my book on Diseases of the Ovaries (4th edition, 1883), I say: "Dr. Battey, of Rome, Ga., successfully operated upon a patient suffering from serious and complicated symptoms. Dr. Battey was the first to publish his proceedings in the ATLANTA MEDICAL JOURNAL (September, 1872), whilst I contented myself with discussing the principles only in my Hastings essay on Diseases of the Ovary (published in 1873, but written in 1872.")

Dr. Battey has in turn done me an injustice which is probably due to a lack of recollection on his part of what took place whilst he was my guest here from July 27 till August 3, 1881, and also what took place at the meeting of the International Medical Congress in London, on August 5th, of the same year.

On the morning of August 2, 1881, I drove Dr. Battey to see a patient from whom I had removed a small ovary for obstinate and intense pain on February 11, 1872, and I brought from a distance of forty miles the patient whom he says died after the operation on August 1, 1872, in order that he might see for himself the object of this strange mistake, and of the first successful operation for removal of the uterine appendages for the treatment of bleeding myoma.

At the meeting of the International Medical Congress, on August 5th, when Dr. Battey read his paper, I distributed a list of all the cases I had done up to the 14th of July, 1881, and I enclose now a copy of that list from the same bundle as those distributed (to the number of two hundred and fifty) in the room. Dr. Battey had a copy of that list.

At the meeting I specially drew attention to Case II. on the list, because it had by error been published as a fatal case in the *British Medical Journal* for 1879, and I explained how the strange mistake had occurred—a kind of mistake which certainly no one could be accused of making intentionally. I need not say that I was surprised, therefore, when Dr. Battey's paper came to be published, a few months after, that he should not have made the correction which he knew was to be justified, and which he ought to have made. Although he had himself seen and questioned the living patient nine years after her operation, and I remember well he satisfied himself of the complete arrest of menstruation as an immediate result of the operation, his paper said: "August 1, 1872, Lawson Tait practiced it at Birmingham with a fatal result." Dr. Marion Sims saw the patient at the same time, and, I think, so did Dr. Briggs, of Nashville, and Dr. Roselmyl, of Hamilton.

Finally, Dr. Battey is wholly in error when he says that my voice was not heard from September, 1872, to May, 1879, on this subject. My first utterance will be found, as I say, in my Hastings essay, published in 1873, and delivered under seal a long time before its publication, and in my own neighborhood and elsewhere I advocated the removal of the uterine appendages for actual disease, but not for the reflex conditions advocated by Dr. Battey. Battey's operation I did not do till August 11, 1879, and now I do not do it at all. On August 2, 1881, Dr. Battey saw me operate for hydrosalpinx—I think the first case he ever saw. But between 1872 and 1878 were perilous times. The clamp in ovariotomy kept us in terror with a mortality of 25 per cent., and no advance was possible. As soon as we were freed from this, we went ahead freely, and now the mortality of all our abdominal operations, hysterectomy alone excepted, has practically vanished.

Priority, after all, is an issue perfectly immaterial, but the mixing up of the principles advocated by Dr. Battey and those advocated by myself, as belonging to the same operation, is an error against which I shall never cease to protest.

I am, etc., Lawson Tait.

Birmingham, June, 1886.

